# Using Mechanical Turk to recruit participants for internet intervention research: experience from recruitment for four trials targeting hazardous alcohol consumption

**DOI:** 10.1186/s12874-017-0440-3

**Published:** 2017-12-01

**Authors:** John A. Cunningham, Alexandra Godinho, Vladyslav Kushnir

**Affiliations:** 1 Centre for Addiction and Mental Health, 33 Russell St., Toronto, Ontario M5S 2S1 Canada; 2 Department of Psychiatry, University of Toronto, Toronto, M5T 1R8 Canada; 3 Dalla Lana School of Public Health, University of Toronto, Toronto, M5S 1A1 Canada; 4 Leslie Dan Faculty of Pharmacy, University of Toronto, Toronto, M5S 3M2 Canada

**Keywords:** Amazon Mechanical Turk, Internet, Online web, Data collection, Research methods

## Abstract

**Background:**

Mechanical Turk (MTurk) is an online portal operated by Amazon where ‘requesters’ (individuals or businesses) can submit jobs for ‘workers.’ MTurk is used extensively by academics as a quick and cheap means of collecting questionnaire data, including information on alcohol consumption, from a diverse sample of participants. We tested the feasibility of recruiting for alcohol Internet intervention trials through MTurk.

**Methods:**

Participants, 18 years or older, who drank at least weekly were recruited for four intervention trials (combined sample size, *N* = 11,107). The same basic recruitment strategy was employed for each trial – invite participants to complete a survey about alcohol consumption (less than 15 min in length, US$1.50 payment), identify eligible participants who drank in a hazardous fashion, invite those eligible to complete a follow-up survey ($10 payment), randomize participants to be sent or not sent information to access an online intervention for hazardous alcohol use. Procedures where put in place to optimize the chances that participants could only complete the baseline survey once.

**Results:**

There was a substantially slower rate of recruitment by the fourth trial compared to the earlier trials. Demographic characteristics also varied across trials (age, sex, employment and marital status). Patterns of alcohol consumption, while displaying some differences, did not appear to vary in a linear fashion between trials.

**Conclusions:**

It is possible to recruit large (but not inexhaustible) numbers of people who drink in a hazardous fashion. Issues for online intervention research when employing this sample are discussed.

## Background

Recruiting participants can be challenging, whether for longitudinal studies, single occasion surveys, or for intervention trials. Many researchers have turned to online advertisement as a means of speeding up participant recruitment. This method of recruitment is particularly useful when the research itself can be conducted online, without requiring face-to-face interaction with the participant. Such online recruitment methods have included use of online newspapers advertisements, Google AdWords, and Facebook, each of which have their respective costs, and vary in the speed with which participants are recruited [1–3].

Another version of online recruitment is via services such as Amazon’s Mechanical Turk (MTurk; www.MTurk.com). MTurk is an online platform where more than half of a million people (primarily restricted to the USA) have signed up as ‘workers.’ These participants then access a list of jobs (including surveys) that they can complete for pay (transferred through the MTurk portal). Individuals, businesses, or researchers who post jobs for completion, referred to as ‘requesters,’ are only privy to each worker’s MTurk ID number, and with the survey being conducted on a server completely separated from the MTurk portal (i.e., Amazon cannot access the data collected by the researcher), it allows for a situation of near anonymity for the recruited participants. It is this anonymity, along with the potential to find another means of recruiting large numbers of participants quickly and at low cost, that makes MTurk potentially attractive for online intervention trials. The MTurk portal has already been used for many online research studies [4–9]. However, few of these studies have employed longitudinal designs [10] and there appears to be limited research employing MTurk participants in intervention trials [6, 11].

Given that existing research has indicated that there are many MTurk participants who drink in a hazardous fashion [10, 12], we sought to determine the utility of this recruitment method in the conduct of intervention trials of Internet interventions for hazardous alcohol consumption. Having a source of readily accessible (and cheaply recruited) participants would be particularly useful for online intervention research as it would allow the evaluation of multiple versions of interventions during the development phase of a project.

### Aims and predictions

We sought to:

1. Design and test recruitment procedures that minimize the chances that participants’ could identify the purpose of the recruitment (beyond answering questions about their alcohol consumption) and to prevent participants from taking part in multiple trials.
2. Test the use of MTurk as a means of recruiting large numbers of people who drink in a hazardous fashion.

We predicted that:

1. Participant recruitment rates would be slower in trial 4 than in trial 1.
2. Demographic and drinking characteristics will vary between trials.
3. Participants randomly assigned to receive the hazardous drinking interventions in each of the trials would report lower levels of drinking at follow-up compared to participants who did not receive access to the intervention (note: outcome results not presented here).

## Methods

We have conducted recruitment for four online intervention trials targeting hazardous alcohol consumption using the MTurk platform (Trial 1: September 2016; Trial 2: December 2016; Trial 3: January 2017; Trial 4: March 2017). The first two stages for all recruitments were the same. An advertisement was placed on the MTurk platform for ‘workers’ to complete a task (called a ‘HIT’ – Human Intelligence Task; in this case to complete a survey), “The Centre for Addiction and Mental Health is conducting a survey on people’s drinking. Only people who currently drink alcohol are asked to participate.” The workers were told that the survey would take less than 15 min to complete and that the payment was $1.50 (an amount in line with other surveys advertised on MTurk; note that Amazon charges an extra 40% on top of this payment for use of the MTurk platform). Workers who clicked on the link were taken to a brief description of the study (a survey on people’s drinking) and were asked to complete a screener to determine eligibility (must be 18 years or older, report drinking once per week or more often); the study description and brief eligibility screener (along with the rest of the trial materials) were located on a separate survey that was independent of the MTurk portal. Those who were eligible were sent to an online consent form that described the purpose of the study (people’s experiences with drinking). This consent form mentioned that some participants would be asked if they would like to take part in another server but that we did not know if they would be asked as of yet (setting up for the recruitment of eligible participants for the intervention trials). Those who agreed to participate were then sent to the online baseline survey.

Those participants who completed the baseline recruitment and accurately answered the attention check questions (see details in next section) comprise the baseline survey recruitment samples discussed in this manuscript. The recruitment methods for all 4 trials were identical, allowing for comparisons of rate of recruitment, and participant characteristics between the four trials. We captured the MTurk worker number for all participants at stage 1 of recruitment, and then ensured that MTurk workers could only complete the eligibility screener once throughout the recruitment for the four trials. Participants identified as hazardous drinkers (Trial 1: AUDIT score of 8 or more; Trials 2-4: Audit score 8 or more and typically consume 15 or more drinks per week) were then invited to take part in another study (Proportion [n] of baseline survey sample eligible and consenting to participate in each intervention study: Trial 1 = 48.6% [423]; Trial 2 = 30.9 [1003]; Trial 3 = 28.8% [996]; Trial 4 = 25.1% [887]).

### Advantageous details of the MTurk platform when conducting multiple recruitments

When conducting multiple studies from the same account, the MTurk platform allows the requestor (the customer hiring the workers) to assign workers who have completed a HIT a code (a ‘qualification’; specific to one requestor’s account). This then allows requestors to restrict subsequently posted HITs to workers who have not been assigned a code; thus preventing workers who do not meet screener criteria from reattempting the survey during another day of recruitment as they are prevented from seeing the HIT advertisement again. The same process can be used to ensure that workers only take part in one of multiple trials. As can be seen on Table 1, which lists the data cleaning steps for the baseline surveys on all four trials, the assignment of qualifications is not a perfect solution. For example, qualifications cannot be assigned to workers who complete the survey, but do not submit the HIT, or to workers who only partially complete a survey. However, post-hoc data cleaning allows the researcher to ensure that each worker is only included once in the set of studies (assuming that the same participant does not have several MTurk ID numbers – this is hard to accomplish as Amazon collects the workers’ Social Security Number on registration).

**Table 1:** Final baseline survey sample size calculation

	Trial 1	Trial 2	Trial 3	Trial 4
Duration of trial recruitment	3.2 h	7 days	9 days	32 days
Total # (*N*) of surveys accessed	1252	4943	5412	5846
Total # of surveys removed % (of *N*)	30.0 (381)	34.4 (1699)	36.1 (1956)	39.5 (2310)
# (*n*) Reasons for removal [% of *n*]	381	1699	1956	2310
Blank surveys	1.0% (1)	1.9% (33)	1.9% (38)	1.5% (34)
Duplicates and suspicious data ^a^	9.2% (35)	11.8% (200)	3.6% (71)	9.5% (220)
Incomplete eligible screeners	8.9% (34)	2.8% (47)	4.1% (81)	5.4% (125)
Ineligible screeners ^b^	50.7% (193)	54.3% (923)	65.6% (1284)	58.8% (1359)
Participants did not pass all attention checks	27.8% (106)	27.4% (466)	22.1% (432)	21.0% (485)
Participants who indicated they did not respond honestly	2.4% (9)	1.8% (30)	2.6% (50)	3.8% (87)
Final baseline survey sample size	871	3244	3456	3536

^a^ Participants who accessed the survey multiple times, but only submitted one complete survey were retained once in the final sample. Those who completed the entire survey more than once were considered to have provided suspicious data and are not included in the final sample size

^b^ Ineligible screeners refers to those who did not proceed to the baseline survey (i.e. were <18 and reported consuming alcohol less than once per week)

In order to handle the processing of participants (e.g., paying participants, sending further information to those eligible and agreeing to take part in an intervention trial), it sometimes makes sense to restrict the number of participants recruited on each day, particularly when the recruitment rate is fast (such as with the first two studies). It is also recommended to restrict the survey to good quality participants who have completed at least 100 HITs and received an approval rating of 95% (i.e., fewer than 5% of the tasks/survey completed resulted in a disapproval rating from the requestor advertising the HIT) [13]. Other steps recommended to optimize the collection of good quality data are to include attention check questions and to ask the participant if they provided honest responses (while emphasizing that their response will not impact on their payment or approval rating) [13]. The interested reader can find more tips regarding the use of MTurk in the extant literature (e.g., [6]). Finally, because our interventions were optimized for use in North America, we restricted participants to those who reported living in the US or Canada (note that at the time these trials were conducted, there were very few Canadian MTurk workers).

### Ethical approval

The research methods to be used in this study have been approved by the standing ethics review committee of the Centre for Addiction and Mental Health (CAMH).

### Survey content

All four recruitment surveys contained a core set of items (with some additional items added as we progressed to the later studies). Demographic characteristics collected were age, sex, marital and employment status, family income, and ethnic origin. Later surveys also included country of residence and number of HITs completed as an MTurk worker. The drinking items consisted of the Alcohol Use Disorders Identification Test (AUDIT) [14], quantity consumed on each day of a typical week, highest number of drinks consumed on one occasion, perceived risk associated with drinking (10-point scale from no risk to high risk), experience of consequences (10 items adapted from Wechsler et al., [15] with one item added asking about driving under the influence of alcohol), and use of alcohol-related treatment services (single item screener taken from the National Epidemiological Survey on Alcohol and Related Conditions) [16]. For the alcohol quantity and frequency items, and the consequence items, the questions were framed to refer to either the past 3 months (for the first study), or past 6 months (for the last three studies). This allowed for the assessment of consumption and consequences using the same time frame as that of the follow-up survey completed in the intervention trial (3 months in the first study and 6 months in the remaining studies).

### Data analysis

In addition to summarizing recruitment numbers (and numbers removed) in Table 1, the analyses consist of bivariate comparisons of demographic and drinking characteristics between the four trial recruitments, and a Poisson regression to compare the rate of recruitment between the four trials. For the Poisson regression, time of recruitment for each trial was rounded up to number of days.

## Results

The combined data set contained a total of 11,107 participants. As can be seen on Table 1, there was a general trend towards the necessary removal of more surveys as part of later recruitment efforts versus the earlier ones. As for the reasons for discarding surveys, the only category that displayed a consistent increase across recruitments was that more prospective participants appeared to be screened out at the earliest stages of the recruitment process (prior to accessing the consent form). In addition, a Poisson regression demonstrated that the rate in which participants were recruited decreased across the four trials (rounded up to number of days), as the rate of recruitment in trial 1, 2 and 3 was 7.9 (95% CI, 7.3 to 8.5) times, 4.2 (95% CI, 4.0 to 4.4) times and 3.5 (95% CI, 3.3 to 3.6) times higher than the rate of recruitment for trial 4, respectively.

There were some minor demographic characteristic differences between trials (see Table 2). For trials where data was available, it was found that almost all participants resided in the US (Trials 3 and 4; 98.8 – 99%) and had completed a median of 500 HITS on MTurk (Trial 4). Participants’ age differed across the trials (F[3,11,103] = 29.1, *p* < 0.001). Post hoc analyses using Bonferroni corrections (or Games-Howell tests when variances were unequal) revealed that participants in trial 4 were more likely to be younger than those in the earlier three trials (*p* < 0.001, for all three) and participants in trial 3 were more likely to be younger than those in trial 1 and 2 (*p* = 0.001, *p* < 0.001, respectively). There also appeared to be a lower proportion of males, those who were married or common law, and full-time employed participants in the later trials compared to the earlier ones (*X* ^2^ [3] = 17.3, *p* = 0.001, *X* ^2^ [3] = 13.7, *p* = 0.003, and *X* ^2^ [3] = 74.0, *p* < 0.001, respectively). Lastly, participants in trial 1 also appeared significantly more likely to report that their family income was less than US$20,000 compared to all other three trials (*X* ^2^ [3] = 47.8, *p* < 0.001). Finally, ethnicity (% white), while significantly different (*p* < .05), did not display a consistent trend across trials.

**Table 2:** Demographic characteristics of the four recruitment samples

	Trial 1 (*n* = 871)	Trial 2 (*n* = 3244)	Trial 3 (*n* = 3456)	Trial 4 (*n* = 3536)	*p*
US Resident, % (*n*)	–	–	99.0 (3421)	98.8 (3492)	0.360
Mean (SD) Age	36.4 (10.5)	35.5 (10.9)	34.5 (10.7) ^a, b^	33.5 (10.4) ^a, b, c^	<0.001
Male, % (*n*)	47.9 (417)	46.8 (1516)	42.8 (1478) ^a, b^	43.2 (1527) ^b^	0.001
White, % (*n*)	82.9 (722)	80.6 (2615)	79.1 (2735)	82.3 (2910) ^c^	0.003
Some post-secondary education, % (*n*)	72.9 (635)	71.1 (2305)	72.0 (2489)	71.2 (2517)	0.618
Married / Common-law, % (*n*)	53.7 (468)	50.1 (1624)	48.1 (1661) ^a^	47.5 (1679) ^a^	0.003
Full-time employed, % (*n*)	70.3 (612)	64.7 (2098) ^a^	59.7 (2062) ^a, b^	57.2 (2022) ^a, b^	<0.001
Family income less than US$20000, % (*n*)	27.6 (240)	17.3 (560) ^a^	19.3 (667) ^a^	20.5 (724) ^a, b^	<0.001

Group differences were computed using chi-squares and one-way ANOVA tests. To determine specific group differences, post-hoc tests were performed with Bonferroni corrections; ^a^ significant difference compared to Trial 1; ^b^ significant difference compared to Trial 2; ^c^ significant difference compared to Trial 3

Bivariate comparisons revealed some small differences across trials in drinking measures and treatment access (see Table 3). Overall, mean AUDIT scores were observed to be significantly lower in trial 1 compared to all other trials, however a general decrease in AUDIT scores reported between participants was observed across trials 2 to 4 (*F*(3, 11,103) = 16.9, *p* < 0.001). In addition, participants in trial 1 also reported consuming significantly fewer drinks on one occasion, experienced fewer consequences from their drinking, and were less likely to have ever attended treatment compared to participants in the other trials, *F*(3, 10,944) = 18.4, *p* < 0.001, *F*(3, 11,103) = 25.7, *p* < 0.001, and *X* ^2^(2) = 8.4, *p* = 0.015, respectively.

**Table 3:** Clinical and drinking characteristics of the four recruitment samples

	Trial 1 (*n* = 871)	Trial 2 (*n* = 3244)	Trial 3 (*n* = 3456)	Trial 4 (*n* = 3536)	*p*
AUDIT, mean (SD)	9.3 (6.6)	10.9 (7.3)^a^	10.8 (7.3)^a^	10.1 (6.9)^a, b, c^	<0.001
Number of drinks consumed in a typical week, mean (SD) ^d^	5.1 (1.9)	6.0 (1.8)^a^	6.0 (1.8)	5.9 (1.8)^b, c^	<0.001
Largest number of drinks consumed on one occasion, mean (SD) ^d^	8.4 (2.3)	9.3 (2.4)^a^	8.8 (2.4)^a^	8.3 (2.4)^a^	<0.001
Number of consequences experienced, mean (SD)	1.7 (1.8)	2.3 (2.1)^a^	2.4 (2.1)^a^	2.2 (2.0)^a, c^	<0.001
Ever attended formal treatment, % (*n*)	7.0 (61)	10.0 (323)^a^	10.2 (352)^a^	–	0.015

*AUDIT* Alcohol Use Disorders Identification Test

Group differences were computed using chi-squares and one-way ANOVA tests. To determine specific group differences, post-hoc tests were performed with Bonferroni and Games-Howell corrections; ^a^ significant difference compared to Trial 1; ^b^ significant difference compared to Trial 2; ^c^ significant difference compared to Trial 3

^d^ Geometric means were used to account for the positive skew observed in the raw data; computed by averaging logarithmic values, and then converted back to a base 10 number of drinks

## Discussion

It was possible to recruit large numbers of participants who drank in a hazardous fashion. In addition, many were interested in completing another survey (for a $10 payment) and, at least for the first trial, the follow-up rate was good (85%) [17]. However, MTurk does not contain an inexhaustible supply of participants who drink in a hazardous fashion [6]. After recruiting for multiple trials in a short time period, the pace at which participants were recruited to complete the baseline survey reduced noticeably, leading to recruitment phases that lasted weeks rather than hours to complete. There were also larger numbers of participants who got screened out of the recruitment process prior to completing the baseline survey.

There are a variety of different methods that have been employed to recruit participants for online intervention trials. The experience of recruitment (speed, participant characteristics) will no doubt vary by country within which the study is conducted (e.g., due to factors such as population size and proportion of the population that accesses the Internet) and the recruitment advertisement employed. However, the first author has employed different recruitment methods for online trials within Canada largely, allowing for some descriptive comparisons (although there is still the substantial limitation that the conduct of these studies have spanned almost a decade). Our primary observation is that the characteristics of the MTurk recruited population more closely match the drinking patterns of participants recruited through general population surveys [18] as compared to recruitment through paper newspapers [19] or through online recruitment (e.g. newspaper website, Google AdWords, Facebook) [20]. Specifically, when using a recruitment cut-off of an AUDIT score of 8 or more, there appears to be a larger proportion of participants who report less severe alcohol use (i.e., lower AUDIT scores) and low levels of typical weekly alcohol consumption by participants recruited from both MTurk and general population surveys compared to participants recruited through paper advertisements or through other online advertisement sources. We were also able to recruit participants more quickly using MTurk than in our other trials using alternate advertising methods (although it is important to note that other researchers have been able to obtain study samples quickly using Facebook or Google AdWords and that the recruitment on MTurk was substantially slower by the forth trial compared to earlier trials) [21].

There were some systematic patterns in demographic characteristics between trials 1 through 4 [6]. Participant age was lower in trial 4 compared to trial 1, as was the proportion of males, being married or common/law, or full-time employed. With the exception of employment status, none of the demographic characteristics varied more than 10%. Nevertheless, taken together, these variations in demographic characteristics indicate that the samples recruited from trial 1 to 4 cannot be taken to be random samples from a large pool of participants but rather reflect something more like a changing sample that occurs as eager (or easily accessible) participants are recruited followed by people who would be progressively less likely to quickly choose to respond to a survey about alcohol use. This may be akin to the phenomenon seen in samples generated for random digit dialling telephone surveys, where those contacted late in the recruitment process (and after a progressively larger number of contact attempts) have systematically different characteristics than those who were contacted earlier [22, 23]. Unlike demographic characteristics, there did not seem to be large (or systematic changes) in drinking characteristics, with small increases in Trial 2 and 3 followed by minor reductions at time 4. These changes may more likely be the result of the time of year the recruitment occurred (December and January for Trials 2 and 3) rather than a change represented by having exhausted quickly responding participants.

There are some strengths and weaknesses associated with recruiting participants for alcohol intervention trials (or other longitudinal studies) using MTurk. Limitations include the recruitment of samples that are very experienced with filling out surveys and who are most likely largely taking part in the trial for financial reasons rather than for concern about their drinking [6]. While this could also be the case for other trials using different recruitment methods (whether college samples or by other types of online recruitment), the ‘contract’ involved is explicit, making it hard to ignore for both the researcher conducting the study and for others reading the resulting articles. Further, unless the researcher is careful regarding what type of information is provided about the desired participants for the trial, it is possible that participants could provide responses to increase their chances of being included rather than being an accurate reflection of their drinking [6]. Offsetting these possible demand characteristics, and if the recruitment process is conducted carefully, the fact that the researcher has almost no personally identifying information from participants (only the participants’ MTurk worker ID number), could actually lead to more accurate self-reports regarding drinking [24].

Beyond the accuracy of drinking self-reports, there are other potential limitations resulting from the participants’ reactions to the intervention. Assuming the participants do drink in a hazardous fashion, they have not explicitly signed up for an intervention trial to reduce their drinking. This does have the advantage of allowing for designs where some participants are not provided with an intervention at all (and further, had no expectation of receiving an intervention) [25]. However, for those who do receive the intervention, how do they use it? Are they interested and engaged in the intervention with the goal of addressing their drinking? The motivational characteristics of participants in intervention trials recruited using MTurk almost certainly vary from those characteristics of participants accessing the same interventions when they are seeking help. While this is the same situational dynamic that may occur in other intervention research, the extent to which these dynamics are explicit in a situation where ‘workers’ are hired from MTurk emphasizes the limitations of the generalizability of any results observed and the need for caution regarding statements as to the effectiveness of the intervention in a real world setting.

## Conclusions

It appears possible to set up a recruitment method in MTurk where participants can be screened for eligibility to take part in an alcohol Internet intervention trial without there being a high likelihood that participants will distort their responses in order to meet eligibility criteria. Further, it is possible to recruit large numbers of participants within days, at least when initially employing MTurk as a participant source. However, it is important to recognize that MTurk workers responses to an alcohol Internet intervention may not be generalizable to the intended target audience of these interventions, and that the workers are largely experienced survey completers who are participating for pay.

## Abbreviations

AUDIT: Alcohol Use Disorders Identification Test
MTurk: Mechanical Turk

## References

[1] Gross MS, Liu NH, Contreras O, Munoz RF, Leykin Y (2014). Using Google AdWords for international multilingual recruitment to health research websites. J Med Internet Res.

[2] Thornton LK, Harris K, Baker AL, Johnson M, Kay-Lambkin FJ (2016). Recruiting for addiction research via Facebook. Drug Alcohol Rev.

[3] Whitaker C, Stevelink S, Fear N (2017). The use of Facebook in recruiting participants for Health Research purposes: a systematic review. J Med Internet Res.

[4] Shapiro D, Chandler J, Mueller PA (2014). Using mechanical Turk to study clinical populations. Clin Psychol Sci.

[5] Buhrmester M, Kwang T, Gosling SD (2011). Amazon's mechanical Turk: a new source of inexpensive, yet high-quality, data?. Perspect Psychol Sci.

[6] Chandler J, Shapiro D (2016). Conducting clinical research using Crowdsourced convenience samples. Annu Rev Clin Psychol.

[7] Daly TM, Nataraajan R (2015). Swapping bricks for clicks: crowdsourcing longitudinal data on Amazon Turk. J Business Res.

[8] Litman L, Robinson J, Abberbock T. TurkPrime.Com: a versatile crowdsourcing data acquisition platform for the behavioral sciences. Behav Res Methods. 2017;49(2):433–442. 10.3758/s13428-016-0727-z PMC5405057 27071389

[9] Wiens TK, Walker LJ (2015). The chronic disease concept of addiction: helpful or harmful?. Addict Res Theory.

[10] Kim HS, Hodgins DC (2017). Reliability and validity of data obtained from alcohol, cannabis, and gambling populations on Amazon's mechanical Turk. Psychol Addict Behav.

[11] Bunge EL, Williamson RE, Cano M, Leykin Y, Munoz RF (2016). Mood management effects of brief unsupported internet interventions. Internet Interv.

[12] Kristan J, Suffoletto B (2015). Using online crowdsourcing to understand young adult attitudes toward expert-authored messages aimed at reducing hazardous alcohol consumption and to collect peer-authored messages. Transl Behav Med.

[13] Peer E, Vosgerau J, Acquisti A (2014). Reputation as a sufficient condition for data quality on Amazon mechanical Turk. Behav Res Methods.

[14] Saunders JB, Conigrave KM (1990). Early identification of alcohol problems. CMAJ.

[15] Wechsler H, Davenport A, Dowdall G, Moeykens B, Castillo S (1994). Health and behavioral consequences of binge drinking in college: a national survey of students at 140 campuses. JAMA.

[16] Grant BF, Moore TC, Shepard J, Kaplan K (2003). Source and accuracy statement. Wave 1. National Epidemiologic Survey on alcohol and related conditions (NESARC).

[17] Cunningham JA, Godinho A, Kushnir V (2017). Can Amazon's mechanical Turk be used to recruit participants for internet intervention trials? A pilot study involving a randomized controlled trial of a brief online intervention for hazardous alcohol use. Internet Interv.

[18] Cunningham JA, Wild TC, Cordingley J, van Mierlo T, Humphreys K (2009). A randomized controlled trial of an internet-based intervention for alcohol abusers. Addiction.

[19] Cunningham JA (2012). Comparison of two internet-based interventions for problem drinkers: randomized controlled trial. J Med Internet Res.

[20] Cunningham JA, Shorter GW, Murphy M, Kushnir V, Rehm J, Hendershot CS (2017). Randomized controlled trial of a brief versus extended internet intervention for problem drinkers. Int J Behav Med.

[21] Riper H, Blankers M, Hadiwijaya H, Cunningham J, Clarke S, Wiers R, Ebert D, Cuijpers P (2014). Effectiveness of guided and unguided low-intensity internet interventions for adult alcohol misuse: a meta-analysis. PLoS One.

[22] Trewin D, Lee G, Groves RM, Biemer PP, Lyberg LE, Massey W, Nicholls L, Waksberg J (1988). International comparisons of telephone coverage. Telephone survey methodology.

[23] Kandel D. The social demography of drug use. Milbank Quarterly. 1991;69(3):365–414. 1805113

[24] Babor TF, Steinberg K, Anton R (2000). Talk is cheap: measuring drinking outcomes in clinical trials. J Stud Alcohol.

[25] Cunningham JA, Koski-Jannes A, Wild TC, Cordingley J (2002). Treating alcohol problems with self-help materials: a population study. J Stud Alcohol.

